# A Case of Acute Right Coronary Artery Ostial Obstruction Six Days After Surgical Aortic Valve Replacement Requiring Emergent Coronary Artery Bypass Grafting

**DOI:** 10.7759/cureus.63612

**Published:** 2024-07-01

**Authors:** Yojiro Machii, Atsushi Harada, Fumihiro Kitashima, Naoki Eguchi, Masashi Tanaka

**Affiliations:** 1 Department of Cardiovascular Surgery, Nihon University School of Medicine, Tokyo, JPN

**Keywords:** stemi, right coronary artery, ostial obstruction, bypass grafting, aortic valve replacement

## Abstract

Acute coronary artery obstruction after surgical aortic valve replacement (SAVR) is a rare but potentially life-threatening event that must be prevented. Here, we report a rare case of an 84-year-old woman who underwent SAVR with a 19-mm aortic bioprosthetic valve for severe aortic stenosis and who suddenly developed ST-elevation myocardial infarction six days after surgery as a result of right coronary artery (RCA) ostial obstruction. She experienced cardiogenic shock, and mechanical support devices were introduced; however, she underwent emergency coronary artery bypass grafting (CABG) to the RCA (#3) and survived. We were aware of the risk of RCA ostial obstruction intraoperatively but were unable to prevent it because blood flow was preserved in the early postoperative period. The present case is worth reporting because the patient developed fatal STEMI at a time when she would normally be considered for discharge. A major learning point from this case is that a coronary artery ostium that is patent immediately after SAVR may not be sufficient for patients considered at high risk of coronary artery occlusion.

## Introduction

A surgical aortic valve replacement (SAVR) considered successful by surgeons may be immediately offset by postoperative complications. Acute coronary obstruction after SAVR is rare but potentially fatal [1]. Here, we present a rare life-threatening case of sudden obstruction of the right coronary artery (RCA) ostium six days after SAVR and describe its investigation and immediate management. Specifically, the patient in our case developed fatal ST-elevation myocardial infarction (STEMI) at a time when a patient is normally considered for discharge. Therefore, we discuss here the cause and treatment of this case.

## Case presentation

An 84-year-old woman was referred from a previous hospital with dyspnea and chest pain that has persisted for the past three days. Two months prior, an exercise stress electrocardiogram (ECG) performed at the hospital indicated ST-segment depression in the anterior thoracic lead. She had undergone percutaneous coronary intervention for proximal RCA (#1-#2) stenosis 13 years prior; however, all other past medical history was unremarkable.

**Figure 1:**
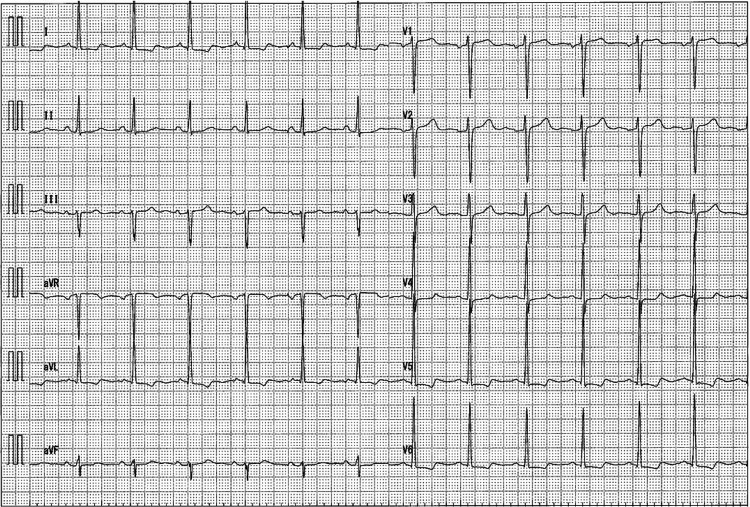
Electrocardiogram on admission There were no obvious ST segment changes.

**Figure 2:**
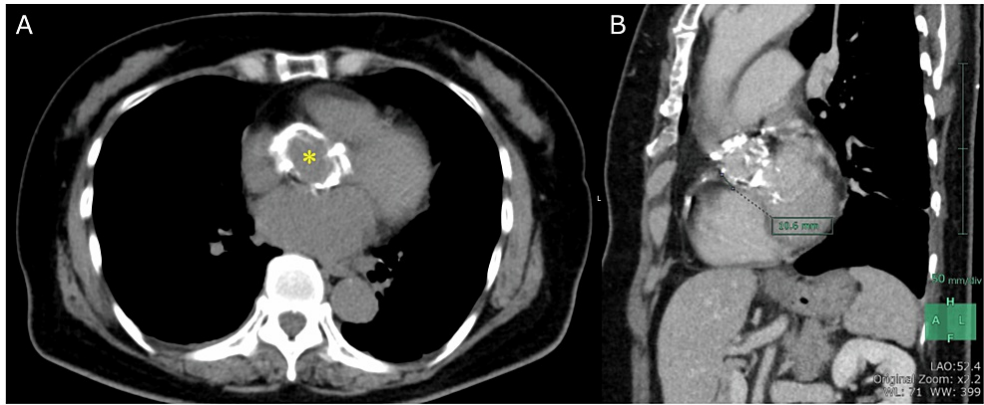
Preoperative chest computed tomography Computed tomography showed severe calcification at the level of the sinus of Valsalva (asterisk) (A). The distance between the right coronary artery ostium and the aortic annulus was 1.06 cm.

On admission, she was 155 cm tall and weighed 51 kg. The body surface area was 1.46 m^2^. Laboratory test results revealed no cardiac enzyme leakages. ECG showed a normal sinus rhythm and no significant ST changes (Figure 1). Transthoracic echocardiography revealed severe aortic valve stenosis (AS) (aortic valve area: 1.06 cm^2^, mean pressure gradient (PG): 53 mmHg) with a normal left ventricular ejection fraction (62%). No other valvular diseases were noted. Transesophageal echocardiography (TEE) revealed severe AS with an annular dimension of 20 mm, a sinotubular junction of 22 mm, and a sinus of Valsalva of 27 mm. Chest computed tomography (CT) showed significant calcification from the aortic root to the ascending aorta (Figure 2A). The distance between the RCA ostium and the annulus was 1.06 cm (Figure 2B). Coronary angiography (CAG) showed no in-stent restenosis in the RCA and no other significant stenosis or collateral vessel development. After discussion with the heart team and providing a detailed explanation to the patient, SAVR was selected instead of transcatheter aortic valve replacement because of the high possibility of valve annular rupture due to severe calcification. 

**Figure 3:**
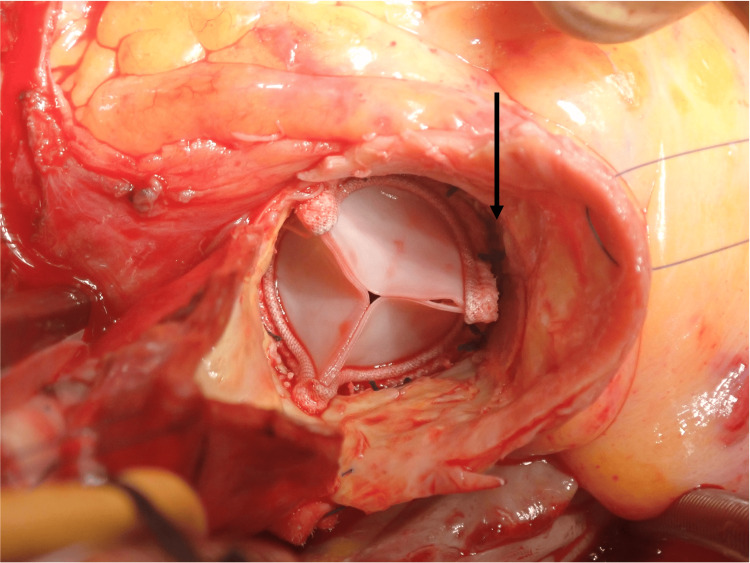
Intraoperative photograph A photograph after the prosthetic valve was implanted; the black arrow indicates the right coronary artery ostium.

Surgery was performed via a median sternotomy. Cardiopulmonary bypass (CPB) was established between the ascending aorta and superior and inferior vena cava. Cardiac arrest was obtained by anterograde through the aortic root and retrograde cardioplegia. Aortotomy was performed approximately 3 cm above the origin of the RCA to avoid calcification. On observation, the aortic valve was tricuspid, with severe calcification from the cusps to the annulus and sinus of Valsalva. The calcifications were removed using a Cavitron Ultrasonic Surgical Aspirator (Cavitron, USA). After confirming the absence of Prosthesis-patient mismatch (effective orifice area index: 1.08 cm^2^/m^2^), we implanted a 19-mm aortic bioprosthetic valve (INSPIRIS RESILIA; Edwards Lifesciences LLC, Irvine, USA). Although the patency of the RCA ostium was confirmed (Figure 3), we performed retrograde coronary perfusion. We determined that the return of the perfusion was insufficient; however, we decided to perform aortic de-clamping because we thought that bypassing RCA would be easier. As a result, she was easily weaned from the CPB, and TEE showed normal right ventricular function; therefore, the operation was terminated without CABG.

**Figure 4:**
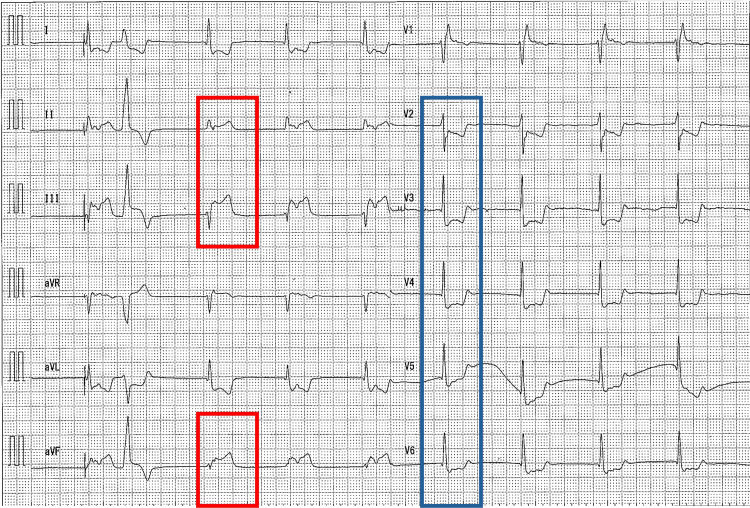
Electrocardiogram at the onset of myocardial infarction Electrocardiogram showing ST-segment elevation in lead II, Ⅲ, and aVF (red), and reciprocal changes in leads V2-V6 (blue).

**Figure 5:**
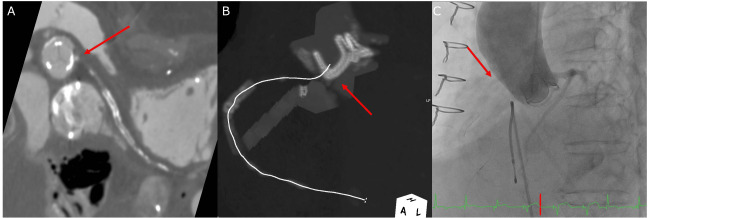
Right coronary artery ostium Coronary computed tomography angiography showing occlusion of the right coronary artery (RCA) ostium (arrow) (A). Maximum intensity projection image showing the RCA ostium close to the stent post of the bioprosthetic valve (arrow) (B). RCA was not contrasted by aortography (C).

The patient’s postoperative course was uneventful. She was extubated the day after surgery, and rehabilitation was initiated. However, on postoperative day (POD) 6, she complained of sudden chest pain and difficulty breathing at midnight. The ECG showed significant ST-segment elevation in the inferior wall, leading to bradycardia (Figure 4). Coronary CT angiography revealed that the RCA ostium was positioned to overlap the stent post of the bioprosthetic valve and the RCA was not contrasted (Figures 5A, 5B). An emergency CAG was performed, but the catheter could not be engaged into the RCA ostium, and the RCA was not contrasted by aortography (Figure 5C). Blood test results showed elevated cardiac enzyme levels (creatinine kinase: 1480 U/L, creatinine kinase-MB: 95 U/L) and a prolonged preoperative prothrombin time/international normalized ratio of 2.32. Immediately after that, the patient experienced cardiogenic shock; therefore, percutaneous cardiopulmonary support and an intra-aortic balloon pump were inserted, and on-pump beating coronary artery bypass grafting (CABG) was performed emergently to the RCA (#3) using a saphenous vein graft (SVG).

**Figure 6:**
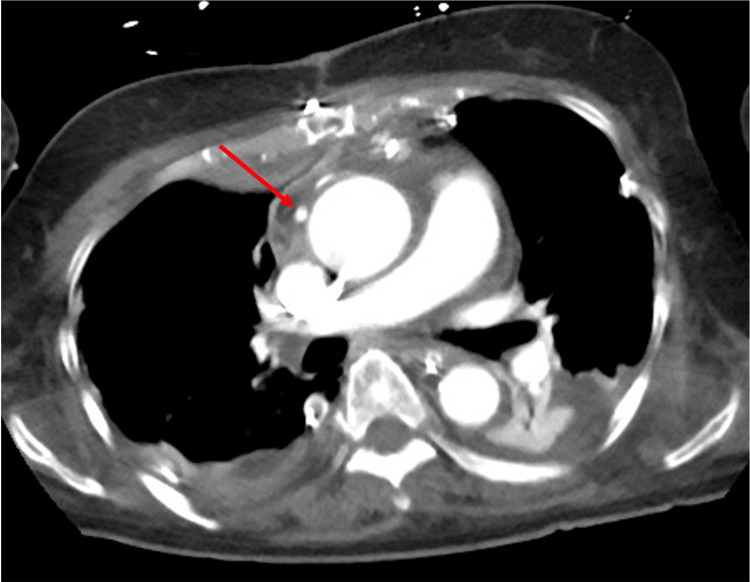
Postoperative contrast-enhanced computed tomography The red arrow indicates saphenous vein graft and the bypass to the right coronary artery was patent.

Postoperative cardiac function improved, and weaning from mechanical support devices was uneventful. There was no increase in the PG across the prosthetic valve (mean PG: 8.3 mmHg). Contrast-enhanced CT confirmed that the SVG was patent (Figure 6). However, weaning from the ventilator was difficult and tracheotomy was required. The patient also developed mediastinitis; therefore, an omental flap transposition was performed on POD 55. Finally, the patient was transferred to another hospital on POD 88 with difficulty weaning from the ventilator. At the time of this report, she is still undergoing rehabilitation at the hospital.

## Discussion

Coronary ostial stenosis is a life-threatening postoperative complication of SVAR, with a reported complication rate of 1-5% in patients undergoing SAVR [2]. Onset has been reported intraoperatively [3,4] and within 3-6 months postoperatively [5]. Shin et al. reported successful bare-metal stent placement for coronary ostial stenosis 22 months after SAVR [2]. We believe that the present case is worth reporting because the complication occurred on POD 6. Particularly, the patient developed fatal STEMI at a time when she would normally be considered for discharge. Thus, the cause and treatment need to be thoroughly discussed.

The possible mechanisms of intraoperative coronary ostial stenosis include improper positioning of the prosthetic valve, inappropriate aortotomy suture, calcific embolus, and spasm of the coronary arteries [3-6]. Mechanical injury or direct trauma to vessels is usually caused by the use of a coronary perfusion cannula, which results in immediate dissection and myocardial infarction [7]. There are reports of late development due to thromboembolism, fibrosis, and intimal thickening of the aortic root caused by turbulent flow around the prosthetic valve, as well as an immune response to the prosthetic valve [2,7]. In the present case, the RCA had no collateral vessels, suggesting that blood flow from the coronary artery ostium was preserved in the early postoperative period. However, on the sixth POD, the patient suddenly developed coronary ostial obstruction. One possible cause of coronary artery ostial occlusion in the early postoperative period, especially in patients with highly calcified aorta as in this case, could be embolization of plaque. Another possible cause is a postoperative change in the morphology of the sinus of Valsalva, which may have led to occlusion of the coronary artery ostium. In any case, we believe that the proximity of the stent post of the prosthetic valve to the coronary artery ostium was one of the major risk factors. We believe that the position of the prosthetic valve should have been reviewed and root enlargement should have been considered. We anticipated the possibility of intraoperative RCA ostium occlusion and our policy was to perform CABG with SVG if an infarct was identified in the RCA region intraoperatively. However, the patient developed STEMI at an unexpected time. Based on this experience, we believe that patients with a narrow aortic annulus, a short distance from the annulus to the coronary artery ostium, or severe calcification, who are expected to have a high likelihood of coronary artery obstruction after SAVR, should undergo aggressive valve root enlargement with close attention to proper prosthetic valve positioning. An insufficient return of retrograde coronary perfusion, as in this case, may be an indicator of future coronary occlusion. However, it is difficult to prevent coronary ostium occlusion completely, and a sufficient follow-up period after SAVR is necessary to respond to fatal complications as soon as possible. If they do occur, prompt revascularization is required.

## Conclusions

We experienced a case of fatal STEMI six days after SAVR surgery that was resolved by emergent CABG. Coronary artery ostial stenosis after SAVR is a complication that can occur from the early to the late postoperative period. In this narrow annulus case, the bioprosthetic valve interfered with the coronary ostium and tended to embolize, which was thought to be a contributing factor to the coronary ostia occlusion. The occurrence of coronary artery ostia occlusion after SAVR should be prevented and monitored, and if it occurs, it should be revascularized as soon as possible.
